# Constraint 鬱 as a Window on Approaches to Emotion-Related Disorders in East Asian Medicine

**DOI:** 10.1007/s11013-012-9300-0

**Published:** 2012-12-20

**Authors:** Volker Scheid

**Affiliations:** EASTmedicine Research Centre, School of Life Sciences, University of Westminster, London, UK

According to widely publicized WHO data “depression” is an epidemic that by 2020 is set to become the second most important cause of disease throughout the world (World Health Organization 2012). Yet, what the WHO identifies as an emerging pandemic requiring a coordinated global response, more critical voices see as the unreflective application of problematic disease labels that fail to understand and therefore help real patient needs (Summerfield 2006). These critics hold that the classification systems that define psychiatric disorders—the *International Classification of Diseases (ICD)* and the *Diagnostic and Statistical Manual of Mental Disorders (DSM)*—are based on self-descriptions of mental states elicited from patients living in advanced capitalist societies and draw on historically specific definitions of disease, evidence, and facts. In that sense, they constitute “western cultural documents par excellence” (Summerfield 2008). Far from objectively assessing mental health they seek to shape specific ways of citizenship and of being a person that, rather conveniently, match the western neoliberal economic order. Displacing modes of dealing with mental health issues that emphasize interpersonal relationships or social and economic causes, depression thus becomes just one more condition that reflects and sustains globally dominant networks of consumption and control in the twenty first century.

Globalization, however, works in many different directions at the same time. Thus, even as DSM and ICD are westernizing the understanding and treatment mental health issues around the world, health care in the West itself is slipping away from the control of biomedicine and is now, de facto plural. Mirroring the increasing importance of Asian economies on the global stage East Asian medicines are amongst the most influential of the many so-called complementary and alternative medicines (CAM) that now compete with biomedicine in the global health care market. Tracing their origins back hundreds or even thousands of years, none of these medicines knew of “depression” in the manner constituted by ICD or DSM before the final decades of the twentieth century. And yet, the physicians that practice these healing arts around the world today offer solutions for depressive disorders based on what they claim to be thousands of years of clinical experience (Yuan 2009).

How East Asian medicine doctors have adapted their traditions to the treatment of this very modern disease and what questions this raises for humanities scholars, clinicians, patients, and regulators are the stories that the four articles collected in this issue take up. But before we begin, we must clear the decks for readers who are not familiar with “traditional medicine” in East Asia and for those who are more accustomed to view them through a contemporary lens.

First, we note that thinking of East Asian medicines as distinct “national” medical traditions is a relatively recent phenomenon and one that is intimately related to the emergence of the nation state. We argue, instead, that the medical practices of East Asia constitute a unified field, albeit a unity that is loose like the “family resemblance” of Wittengenstein’s language games rather than that of a closed system. Such family resemblance is founded in a shared debt to early Chinese medical texts and maintained by an ongoing exchange of knowledge and technology across the region. In relation to the topic at hand, we clearly see this family resemblance in the fact that despite the many explicit and implicit differences of traditional medical practices in China, Japan, and Korea today, they all approach depression by way of the same concept, that of constraint 鬱 (Chin. *yù;* Jap. *utsu*; Kor. *ul*).

Second, we reject the notion of “traditional” medicine in the sense that it is conventionally employed: that is, as a set of medical practices so integrally linked to its origins that, unlike modern science or biomedicine, it is incapable of profound change. What our papers show, instead, are fundamental transformations in knowledge structures and clinical practice in how East Asian medicines deal with emotion-related disorders through a shared focus on constraint. Some of these transformations occur gradually over centuries while others transpire in decades or less. Some of these changes result from the encounter with modern biomedicine but most took place long before that time. Hence, unlike the impact-response model that guides so much modernization theory, where a passive East is spurred to change only through the climatic encounter with a dynamic West, we do not find these more recent transformations to be determinative. Instead, we hold that the key to understanding how physicians today are responding to the clinical problem of depression can only be located in the broader trajectories of East Asian medicine. We emphasize the word “trajectories” because we find multiple developmental trends that shape the history of medicine across the region.

Scheid, in the first paper in this collection, traces the slow process by which the concept of constraint in late imperial China gradually became associated with emotion-related disorders, with liver system pathologies and, at least in the eyes of some physicians, with a very narrow range of specific treatment regimes. This implied a profound re-interpretation of earlier ideas contained in the foundational texts of East Asian medicines that associated constraint specifically with externally contracted pathogens and with the Lungs. Furthermore, these transformations were enacted primarily in southeast China among an elite stratum of physicians and their patients, reflecting locally specific gender identities and notions of constitution, as well as an ambivalence to the role of emotions in human life. These ideas and practices thus do not in any meaningful way represent “China” as either a political, cultural, or historical entity. Yet, their cultural dominance ensured that they became the foundations on which physicians in contemporary China constructed “traditional Chinese medicine” (TCM).

Having moved to Japan between the twelfth and sixteenth centuries, notions of constraint came to dominate medical thinking there, too, though in ways that were at once similar and radically different from the sources they had initially borrowed. Daidoji, in the second paper of this issue, examines multiple strands of these transformations between the seventeenth and twentieth centuries. She convincingly demonstrates that even as contemporary practices share a certain family resemblance with premodern ones, or those in Edo Japan with those in Ming dynasty China, they are not in any narrow sense transforming out of each other. Rather, she uncovers a distinctive change in Kampo practice during the late nineteenth century, whereas earlier transformations emerge as highly contextual synthesis, driven by the idiosyncratic thought styles of individual physicians and the vagaries of Japanese translations of Chinese terms as much as the specific socio-cultural contexts in which such production occurred.

An important characteristic of this context was the increasing turn towards empirical observation as the foundation of medical practice among leading physicians in Edo Japan. This empiricist turn later inspired physicians in Republican China faced with the increasing visibility of medical practices imported from the West. As Karchmer shows in his essay, modernizing Republican era physicians used this “interruption” as an opportunity to move their tradition towards a path that was at one and the same time rooted in western anatomy and the most ancient medical texts from which physicians had significantly departed long since. In doing so, they connected the nerves and ideas about nervous weakness (or neurasthenia), then just as a much a global epidemic as depression is today, to Chinese medical conceptions of the liver and liver disease. Along the way, they conveniently ignored the debt they owed to the post-Song physicians they so despised, who had moved the liver to the forefront of medical thinking in all cases of emotion-related disorders. Likewise, while Chinese medicine physicians in contemporary China have largely abandoned Republican era attempts to create convergences between the Chinese and western medical bodies in favor of delineating clear differences between their respective medical systems, modern interpretations of emotion-related disorders and constraint as predominantly a liver system disorder are a direct continuation of Republican era practices.

In the final essay of this issue, Soyoung Suh examines Korean responses to an emotion-related disorder endemic to contemporary Korea, that of fire illness or *hwa*-*byung* 火病. Also known as *ui*-*hw*-*byung* 鬱火病, which can be translated as “constraint [leading] to fire illness”, the term is redolent of post-Song interpretations of constraint that viewed fire (i.e., heat symptoms) as one of the most common developments of constraint disorders. Yet, the prevalence of the disease in Korea is rooted not in medical transmission but in contemporary popular culture to which both biomedically trained psychiatrists and practitioners of Korean medicine were forced to find new answers. Such attempts invariably raised questions about what was specifically Korean about this disorder and how it related to both biomedical disease categories such as depression and anxiety *and* to traditional disease concepts of emotional disorders imported from China. Suh argues that ultimately such efforts must be seen attempts to define ones identity in a context where the desire for autonomy is enacted in (neo)colonial discursive formations that are both present and absent to the actors Suh observes.

What emerges from the four essays then is not a single internally consistent history whereby indigenous notions of constraint come to be connected to the biomedical disease category of depression. Rather, physicians in China, Japan, and Korea assemble valid responses—valid because peers, patients, researchers, and regulators take them seriously—to newly emergent medical problems through highly contextual processes of synthesis. Yet, we would argue that a thick description of these processes that uncovers all of the various threads that contribute to these assemblages, is only possible through the kind of trans-local *longue durée* approach we have taken here. By trans-local, we mean that all of the practices and transformations we have described here invariably relate themselves to somewhere and something else: to a shared archive of medical texts; to lineages of transmission and scholarship; to established formulas or treatment strategies; to medicinals imported from different places and diagnostic technologies developed at different times. These elements themselves are equally fluid in their composition and forever travel between sites of articulation with scant respect for boundaries, territorial or otherwise. By emphasizing the concept of *longue durée* we acknowledge that what unites all of these transformations is their relation to the concept of constraint and therefore, at an even deeper level, to that of *qi.*

Read together our essays thus insert themselves into a number of different debates. At the most basic level, they sketch the history of constraint in East Asian medicine from a transnational perspective. They reveal that history to proceed along a number of different trajectories all of which point back to a common origin: the appropriation of Han dynasty concepts of constraint by a small group of physicians in post-Song China from where they dispersed, via various routes, across all of East Asia. These notions were successfully articulated and re-articulated into diverse local contexts of practice but only some of these currents carried all the way into the present to successfully articulate constraint with depression. Of these, TCM’s linkage of depression with the concept of liver qi constraint has been by far the most successful synthesis. Indeed, as described by Scheid and Karchmer, it is this linkage that establishes contemporary TCM as the only truly global alternative to psychiatry-based interventions in the treatment of depression. Other contemporary syntheses have, so far, remained local or regional, though this may well change in the future. Supported by the Korean state, Korean physicians are currently engaged in strenuous attempts to globalize their medicine, too. If successful, this would set up a situation in which two articulations that connect constraint to depression but utilize entirely different treatment approaches would compete with each other as well as with psychiatric treatments. Even more interesting, some of the treatment strategies that endure in Korea and Japan to this date belong to currents that have virtually disappeared in China itself.

Such histories are not merely of intellectual interest but have important practical implications. If the universalizing efforts of Chinese and Korean traditional medicines are clearly tied to nationalist agendas, global users of these medicines are motivated by different goals. Sooner or later—and the collection of these essays is already a part of this process—competing histories and legitimizing strategies must come into conflict with each other. For even as TCM promotes itself as an alternative to biomedical psychiatry, the endurance of pre-TCM currents of practice in contemporary Korea challenges TCM’s claims to represent Chinese medicine *tout court*. This, in turn, will draw renewed attention to the many different currents of practice that persist within the Chinese, Korean, and Japanese medical traditions. Not only patients but also clinicians and researchers interested in East Asian medicines will thus have to ask entirely new set of questions: not merely whether or not something works and how one might go about evaluating it, but which of several alternatives one should focus one’s evaluations on. As our stories show, the mere endurance into the present of a given formula, treatment approach or medical current has many different reasons of which clinical effectiveness is but one among others.

On a second level, our essays can also be read as a contribution to our understanding of the much analyzed process of globalization. As noted above, in the domain of cross-cultural psychiatry this is most commonly explored by examining how one specific ethnomedicine, biomedical psychiatry, succeeds in establishing a universally accepted normative discourse, as well as charting accommodation/resistance to that discourse. Attempts to assimilate indigenous disease categories relating to constraint (such a *yu* in China or *hwa*-*byung* in Korea) to ICD and DSM standards certainly fit into these established narratives. Yet, this narrative is challenged by the *de facto* globalization of TCM constraint, which circumvented establishment networks even as it used them to its own advantage. After all, the more patients are labeled as suffering from depression by psychiatrists, the larger the pool of potential patients looking towards TCM for alternative treatment.

Moreover, the regional flow of ideas and practices relating to the notion of constraint within East Asia, beginning centuries before the encounter of local medical traditions with the West challenges the idea that current processes of globalization are categorically different to what went on before. Instead, by highlighting the enduring importance of regional flows to the constitution of East Asian medicines the modern opposition between the local and the global is interrupted. This creates space for escaping from the narratives of accommodation/resistance that define the response/impact model of modernization and for focusing, instead, on the necessary trans-local nature of the emergent syntheses that construct the practices we describe.

Such a refocusing will necessitate of historians and anthropologists to recalibrate their attention to mapping, analyzing, and comparing what allows some trajectories to extend across time and space, while others lead into cul-de-sacs. One factor, certainly, that allowed the TCM concept of liver qi constraint to travel around the world so quickly was its ability to translate a wide range of emotion-related disorders into a single disease category without thereby disconnecting it from the wider discourse of qi that defines East Asian medicines. This is fundamentally different from the work of biomedically oriented researchers in Korea and Hong Kong, whose attempts to insert notions of constraint into the field of psychiatry forced them to surrender such attachments, effectively disconnecting them from East Asian medical practice.

This, then, is the final point we wish to make. Dealing with a range of problems embodied in what is, after all, the same biological body, psychiatrists and East Asian medicine physicians arrive at essentially similar solutions: pharmacotherapy and talking-therapies. Where they differ is in the mobilization of fundamentally different conceptions of the body/person that constitute their medicines trans-locally. In East Asian medicine, as long as illness is grasped via the concept of constraint, such grasping involves an acknowledgement of the reality of qi flow and transformation; and because qi flow transcends the divisions that psychiatrists make between emotions, the mind, and the body their different trajectories can touch without threatening each other’s integrity. They can only converge, however, by one dominating the other, or by creating something entirely new. As the histories collected here demonstrate, such disappearance is the invariable price of any process of synthesis and emergence.
